# A Real-World Multicenter Retrospective Observational Study on Polish Experience with Nintedanib Therapy in Patients with Idiopathic Pulmonary Fibrosis: The PolExNIB Study

**DOI:** 10.3390/jcm12144635

**Published:** 2023-07-12

**Authors:** Sebastian Majewski, Adam J. Białas, Adam Barczyk, Halina Batura-Gabryel, Małgorzata Buchczyk, Anna Doboszyńska, Katarzyna Górska, Luiza Grabowska-Skudlarz, Hanna Jagielska-Len, Agnieszka Jarzemska, Ewa Jassem, Dariusz Jastrzębski, Aleksander Kania, Marek Koprowski, Michał Krawczyk, Rafał Krenke, Katarzyna Lewandowska, Barbara Mackiewicz, Magdalena M. Martusewicz-Boros, Janusz Milanowski, Małgorzata Noceń-Piskorowska, Agata Nowicka, Kazimierz Roszkowski-Śliż, Alicja Siemińska, Krzysztof Sładek, Małgorzata Sobiecka, Tomasz Stachura, Małgorzata Tomczak, Witold Tomkowski, Marzena Trzaska-Sobczak, Dariusz Ziora, Beata Żołnowska, Wojciech J. Piotrowski

**Affiliations:** 1 Department of Pneumology, Medical University of Lodz, 90-153 Lodz, Poland; 2 Department of Pneumonology, School of Medicine in Katowice, Medical University of Silesia, 40-752 Katowice, Poland; 3 Department of Pulmonology, Allergology and Pulmonary Oncology, Poznan University of Medical Sciences, 60-569 Poznan, Poland; 4 Department of Lung Diseases and Tuberculosis, School of Medicine with the Division of Dentistry in Zabrze, Medical University of Silesia, 40-032 Katowice, Poland; 5 Department of Pulmonology, University of Warmia and Mazury in Olsztyn, Pulmonology Hospital, 10-357 Olsztyn, Poland; 6 Department of Internal Medicine, Pulmonary Diseases and Allergy, Medical University of Warsaw, 02-091 Warsaw, Poland; 7 Clinical Department of Lung Diseases, K. Marcinkowski University Hospital, 65-046 Zielona Gora, Poland; 8 Department of Rapid Pulmonary Diagnostics, Kuyavian and Pomeranian Pulmonology Center, 85-326 Bydgoszcz, Poland; 9 Department of Pneumonology, Medical University of Gdansk, 80-211 Gdansk, Poland; 10 Department of Pulmonology, Jagiellonian University Medical College, 30-688 Cracow, Poland; 11 Department of Civilization Diseases and Lung Diseases, John Paul II Specialist Hospital, 31-202 Cracow, Poland; 12 1st Department of Lung Diseases and Respiratory Allergy, Voivodeship Center for Lung Disease Treatment and Rehabilitation, 91-520 Lodz, Poland; 13 1st Department of Lung Diseases, National Tuberculosis and Lung Diseases Research Institute, 01-138 Warsaw, Poland; 14 Department of Pneumonology, Oncology and Allergology, Medical University of Lublin, 20-090 Lublin, Poland; 15 3rd Lung Diseases and Oncology Department, National Tuberculosis and Lung Diseases Research Institute, 01-138 Warsaw, Poland; 16 Department of Tuberculosis and Pulmonology, West Pomeranian Voivodeship Hospital, 70-204 Szczecin, Poland; 17 Department of Allergology, Medical University of Gdansk, 80-211 Gdansk, Poland; 18 Department of Pulmonology, E.J. Zeyland Wielkopolska Center of Pulmonology and Thoracic Surgery, 60-569 Poznan, Poland

**Keywords:** nintedanib, idiopathic pulmonary fibrosis (IPF), real-world data, efficacy, safety, Poland

## Abstract

Nintedanib is a disease-modifying agent licensed for the treatment of IPF. Data on Polish experience with nintedanib in IPF are lacking. The present study aimed to describe the safety and efficacy profiles of nintedanib in a large real-world cohort of Polish patients with IPF. This was a multicenter, retrospective, observational study of IPF patients treated with nintedanib between March 2018 and October 2021. Data collection included baseline clinical characteristics, results of pulmonary function tests (PFTs), and a six-minute walk test (6MWT). Longitudinal data on PFTs, 6MWT, adverse drug reactions (ADRs), and treatment persistence were also retrieved. A total of 501 patients (70% male) with a median age of 70.9 years (IQR 65–75.7) were included in this study. Patients were followed on treatment for a median of 15 months (7–25.5). The majority of patients (66.7%) were treated with the full recommended dose of nintedanib and 33.3% of patients were treated with a reduced dose of a drug. Intermittent dose reductions or drug interruptions were needed in 20% of patients. Over up to 3 years of follow-up, pulmonary function remained largely stable with the minority experiencing disease progression. The most frequent ADRs included diarrhea (45.3%), decreased appetite (29.9%), abdominal discomfort (29.5%), weight loss (32.1%), nausea (20.8%), fatigue (19.2%), increased liver aminotransferases (15.4%), and vomiting (8.2%). A total of 203 patients (40.5%) discontinued nintedanib treatment due to diverse reasons including ADRs (10.2%), death (11.6%), disease progression (4.6%), patient’s request (6.6%), and neoplastic disease (2.2%). This real-world study of a large cohort of Polish patients with IPF demonstrates that nintedanib therapy is safe, and is associated with acceptable tolerance and disease stabilization. These data support the findings of previously conducted clinical trials and observational studies on the safety and efficacy profiles of nintedanib in IPF.

## 1. Introduction

Idiopathic pulmonary fibrosis (IPF) is a chronic, progressive, and irreversible lung disease with an inevitable fatal prognosis. Its pathogenesis involves repeating micro-injuries to the alveolar epithelium with subsequent aberrant wound healing resulting in progressive lung scarring. The natural history of IPF is outlined by a gradual decline in lung function, continuing physical activity limitation, significant impairment of quality of life, and premature death. The course of the disease is difficult to predict with heterogeneous trajectories; nevertheless, the median survival time is 3 to 5 years [1,2,3]. In the last decade, two disease-modifying agents, namely pirfenidone and nintedanib, have been approved for use in IPF. Both drugs have antifibrotic properties and have changed the management of IPF, providing improvements in clinical outcomes [4,5,6]. Currently, antifibrotics are recognized as a standard of pharmacological treatment of IPF [7,8]. However, the initial lack of reimbursement in Poland has led to significant limitations and delays in wide access to antifibrotic therapy for IPF patients [9]. Recent changes in reimbursement policy have resulted in the launch of pirfenidone for patients with IPF in Poland in January 2017 and nintedanib since March 2018 in the frame of the antifibrotic treatment program refunded by the National Health Fund (NHF).

In retrospect, the pharmacological treatment of IPF has evolved considerably. The initial concept of the disease pathobiology as being largely inflammatory led to the use of immunosuppressive treatment. However, the landmark PANTHER study evaluating the use of combination therapy with prednisone, azathioprine, and N-acetylcysteine in IPF highlighted that immunosuppressive therapy was associated with higher rates of hospitalizations and deaths compared with placebo [10]. The pivotal randomized clinical trials (RCTs) with antifibrotics in IPF provided crucial data on efficacy and safety, and led to their license for IPF providing hope for patients and changing the landscape of disease treatment [4,5,6]. However, the follow-up periods of RCTs with antifibrotics lasted only from 52 to 72 weeks [4,5,6]. Furthermore, precise and strict inclusion and exclusion criteria of RCTs limit the generalization of study results and findings to the real-world setting of clinical practice. Therefore, real-world data (RWD) studies provide additional pieces of valuable evidence on the long-term safety and efficacy of pharmacological intervention in broader patient populations.

To date, several RWD studies on the safety and efficacy of nintedanib in heterogeneous IPF populations have been published; nevertheless, the scientific soundness of many of them suffers from the small number of patients included and/or short follow-up periods [11,12,13,14,15,16,17,18,19,20,21,22,23,24,25]. Moreover, data describing the clinical safety and efficacy of long-term nintedanib therapy in Polish patients with IPF are lacking.

PolExNIB was a multicenter, retrospective, observational study collecting clinical data of patients with IPF treated with nintedanib from March 2018 to October 2021 across 16 referral centers in Poland. The study aimed to provide the first long-term real-world Polish data on the safety and efficacy of nintedanib treatment in patients with IPF. This study is complementary to the recently published RWD study on the Polish experience with pirfenidone therapy in patients with IPF (the PolExPIR study) [26].

## 2. Materials and Methods

### 2.1. Study Population

The present work in general replicated the methodology applied in the recently published RWD study on the Polish experience with pirfenidone in IPF [26]. Briefly, a targeted population of patients with IPF treated with nintedanib in the setting of a therapeutic program reimbursed by the National Health Fund (NHF) for patients with the mild-to-moderate disease was evaluated. The main inclusion criteria for the NHF program were a multidisciplinary team diagnosis of IPF according to international guidelines [27], FVC ≥ 50% of predicted value, and transfer factor of the lung for carbon monoxide (TL_CO_) above 30% of the predicted value. The main exclusion criteria from enrolment to the NHF therapeutic program were largely compatible with contraindications to nintedanib as per medicinal product characteristics and included hypersensitivity to nintedanib, to peanut or soya, or any of the excipients, pregnancy, breast-feeding, moderate or severe hepatic impairment, and severe renal impairment. Moreover, subjects with FVC < 50% of predicted value or TL_CO_ ≤ 30% of predicted value were not eligible for enrolment. All patients starting nintedanib were followed up on regular basis with safety and efficacy assessments. A mandatory re-evaluation of pulmonary function tests (PFTs) every 6 months since drug initiation was demanded by the therapeutic program protocol and a disease progression defined as a significant FVC decline (≥10% of predicted value) over the first 12 months or consecutive 6 months intervals has been set as an exclusion criterion from a continuation of nintedanib therapy (drug stop rule). The PolExNIB study enrolled all IPF patients starting nintedanib at participating centers regardless of the duration of therapy. The patient enrolment period was from March 2018 to October 2021. The Ethics Committee of the Medical University of Lodz waived the requirement for approval, as the study was retrospective, and patients’ data were anonymized (decision no. RNN/46/23/KE).

### 2.2. Data Collection

The PolExNIB study retrieved data of patients with IPF treated with nintedanib across 16 referral centers in Poland. The study investigators reviewed the medical records of all the patients receiving nintedanib at the participating sites. Clinical data collection included baseline characteristics, data on diagnosis and previous treatment for IPF, supplemental oxygen use, PFTs, and a six-minute walk test (6MWT). Longitudinal data on PFTs, 6MWT, adverse drug reactions (ADRs), treatment persistence, and survival were also collected up to 36 months post-inclusion. To avoid possible bias in PFT data interpretation related to the use of various reference equations and various reference values, the measurements of FVC and TL_CO_ performed in participating centers were reported as absolute values and then expressed as the percentages predicted using the Global Lung Function Initiative (GLI) reference values [28,29].

### 2.3. Statistical Analysis

Data were analyzed using R software for MacOS (R Core Team, 2019, Vienna, Austria). The Shapiro-Wilk test was used to assess the normality of data distribution. A mean with standard deviation (SD) describes continuous normally distributed data and a median with interquartile range (IQR) describes non-normally distributed data. Absolute numbers and relative frequencies are used to express categorical data. For the longitudinal analysis, all available data were included and analyzed using the Wilcoxon signed-rank test. For the longitudinal efficacy assessment of the functional parameters (FVC, TL_CO,_ and 6MWT) the difference between the initial and the last data available for the analysis was used. The dynamics of PFT results were analyzed as a difference between the timepoints specified for each comparison separately. Similarly to the PolExPIR study, an additional subanalysis of the functional parameter changes in the 6-month intervals was performed. For a subanalysis of efficacy data patients were divided based on the rate of changes in FVC% of predicted (∆FVC) in 6-month intervals into the following groups: significant improvement (∆FVC > 10%), marginal improvement (10% ≥ ∆FVC > 5%), stabilization (+5% ≥ ∆FVC > −5%), marginal decline (−5% ≥ ∆FVC > −10%), and significant decline (∆FVC ≤ −10%). In terms of the rate of changes in TL_CO_% of predicted (∆TL_CO_) in 6-month intervals, patients were divided into the following groups: significant improvement (∆TL_CO_ > 15%), stabilization (+15% ≥ ∆TL_CO_ > −15%), and significant decline (∆TL_CO_ ≤ −15%). The manuscript’s graphs were prepared using GraphPad Prism 9 (GraphPad Software, La Jolla, San Diego, CA, USA).

## 3. Results

### 3.1. Baseline Characteristics

A total of 501 patients were enrolled in the study. Patients were followed on nintedanib treatment for a median of 15 months (7–25.5). Table 1 reports the baseline characteristics of the study participants. The median age of the participants was 70.9 years (65–75.7) with a marked predominance of males (69.6%). Most patients were active or ex-smokers (73.2%); moreover, about 8% of patients were still actively smoking at the time of enrolment for antifibrotic treatment. According to the multidimensional prognostic staging system for IPF, the median Gender, Age and Physiology (GAP) score was 3 (3–4), and the majority of subjects were in stage I of the GAP index [30]. The median time from the first symptoms to IPF diagnosis was 12 months (6–28) and from diagnosis to the initiation of nintedanib treatment was 3 months (1–11). At baseline, the median FVC was 80.2% of predicted (68.5–95) and the median TL_CO_ was 55.9% of predicted (42.2–69). The mean baseline distance covered in the 6MWT was 436.8 m (102.3) (n = 288) with a median of 5% desaturation (2–8) during the test. The majority of patients (75.2%) had not received any disease-targeted therapy before nintedanib initiation, whereas 17.6% of subjects had been treated with pirfenidone in the past.

### 3.2. Tolerability, Safety, and Drug Persistence

**Figure 1:**
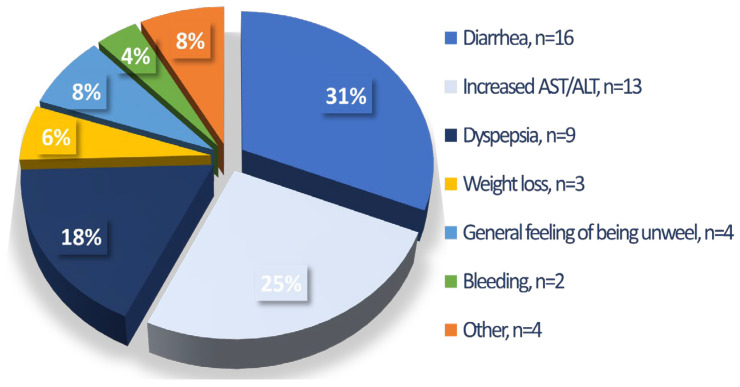
ADRs leading to treatment discontinuation (n = 51). **Abbreviations:** ADRs—adverse drug reactions, AST—aspartate aminotransferase, ALT—alanine aminotransferase. Dyspepsia includes nausea, vomiting, abdominal discomfort, and decreased appetite.

The summary of the tolerability data and ADRs of nintedanib therapy are presented in Table 2. The majority of patients (66.7%) were treated with the full recommended dose of nintedanib (150 mg b.i.d.). A hundred and one patients (20.2%) required dose adjustments including intermittent drug interruption and/or dose reduction to continue treatment adherence, and one-third of patients were treated with a reduced dose of a drug (100 mg b.i.d.). The overall number of patients reporting at least one ADR was 350 patients which accounts for 69.9% of the study cohort. Diarrhea was the most commonly reported ADR (n = 227, 45.3%). Other reported ADRs included decreased appetite (29.9%), abdominal discomfort (29.5%), weight loss (32.1%), nausea (20.8%), fatigue (19.2%), increased liver aminotransferases (15.4%), hepatotoxicity (6.2%), vomiting (8.2%), and bleeding (4%). Over the median time of 15 months (7–25.5) of drug exposure, a total of 203 patients (40.5%) had to permanently discontinue nintedanib treatment due to diverse reasons including ADRs (10.2%), death (11.6%), disease progression (4.6%), patient’s request (6.6%), and neoplastic disease (2.2%), see Table 3. Among the ADRs leading to treatment discontinuation the most common were diarrhea (n = 16, 31%), increased liver aminotransferases (n = 13, 25%), and dyspepsia symptoms (n = 9, 18%) including nausea, vomiting, abdominal discomfort, and decreased appetite, see Figure 1. Over the study period, 72 patients (14.8%) died. IPF-related death occurred in 21 patients (27%), cardiovascular-related death in 3 patients (5.4%), neoplastic-disease-related death in 10 patients (14.9%), coronavirus disease 2019 (COVID-19)-related death in 11 patients (14.9%), and unknown or other cause of death in 27 patients (37.8%).

### 3.3. Efficacy

**Figure 2:**
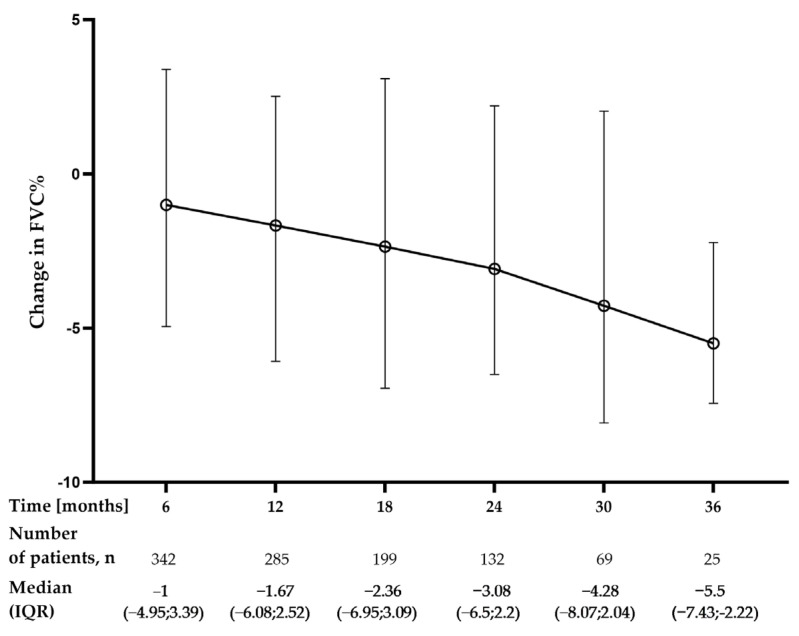
Changes in FVC% of predicted (FVC%) over the study follow-up period. Change from baseline was calculated as a follow-up timepoint value minus the baseline value; therefore, a negative value indicates a decrease from baseline. Data are presented as median (IQR) values. **Abbreviation:** FVC—forced vital capacity.

The longitudinal analysis of PFT data revealed that the median annual decline in forced vital capacity (FVC) during the first year of nintedanib treatment was −60 mL (−220–90) (n = 285) equal to −1.7% of predicted (−6.1–2.4) over the first year, −60 mL (−235–65) (n = 132) equal to −1.6% of predicted (−6–2.1) over the second year, and −100 mL (−250–10) (n = 25) equal to −2.6% of predicted (−7–0.2) over the third year of nintedanib therapy. The median change from baseline in FVC% of predicted was −1.67% (−6.08–2.52) at month 12, −3.08% (−6.50–2.22) at month 24, and −5.5% (−7.43; −2.22) at month 36, see Figure 2.

**Figure 3:**
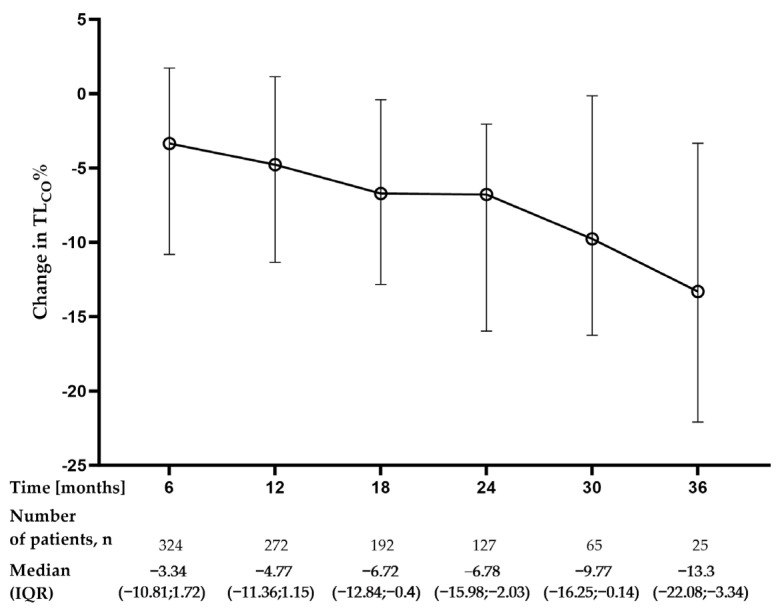
Changes in TLco% of predicted (TLco%) over the study follow-up period. Change from baseline was calculated as a follow-up timepoint value minus the baseline value; therefore, a negative value indicates a decrease from baseline. Data are presented as median (IQR) values. **Abbreviation:** TL_CO_—transfer factor of the lung for carbon monoxide.

The median change from baseline in TL_CO%_ of predicted was −4.77% (−11.36–1.15) at month 12 (n = 272), −6.72% (−15.98; −2.03) at month 24 (n = 127), and −13.3% (−22.08; −3.34) at month 36 (n = 25), see Figure 3.

**Figure 4:**
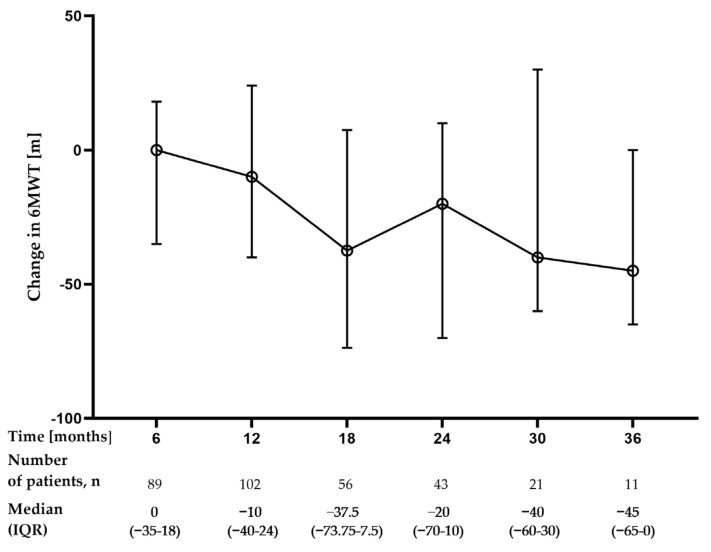
Changes in 6MWT distance over the study follow-up period. Change from baseline was calculated as a follow-up timepoint value minus the baseline value; therefore, a negative value indicates a decrease from baseline. Data are presented as median (IQR) values. **Abbreviation:** 6MWT—six-minute walk test.

The median change from baseline in 6MWT distance during nintedanib treatment was −10 m (−40–24) (n = 102) at month 12, −20 m (−70–10) (n = 43) at month 24, and −45 m (−65–0) (n = 11) at month 36, see Figure 4.

**Figure 5:**
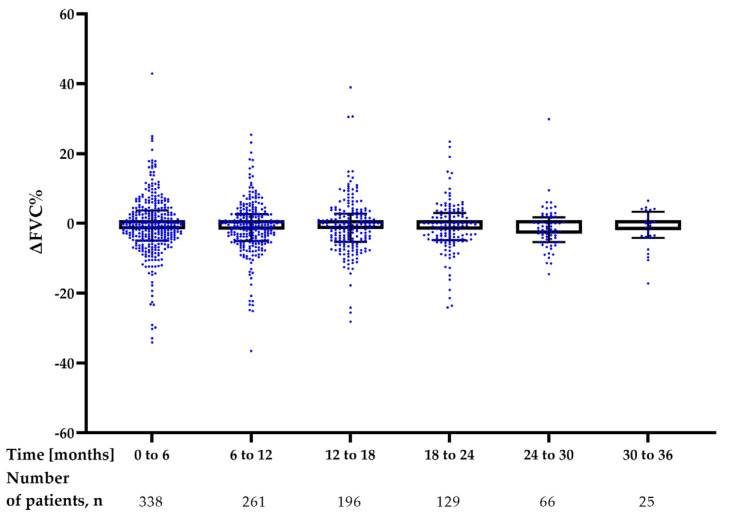
Longitudinal interval changes in FVC% of predicted (ΔFVC%) during nintedanib treatment. Data are presented as median (IQR) values. **Abbreviation:** FVC—forced vital capacity.

**Figure 6:**
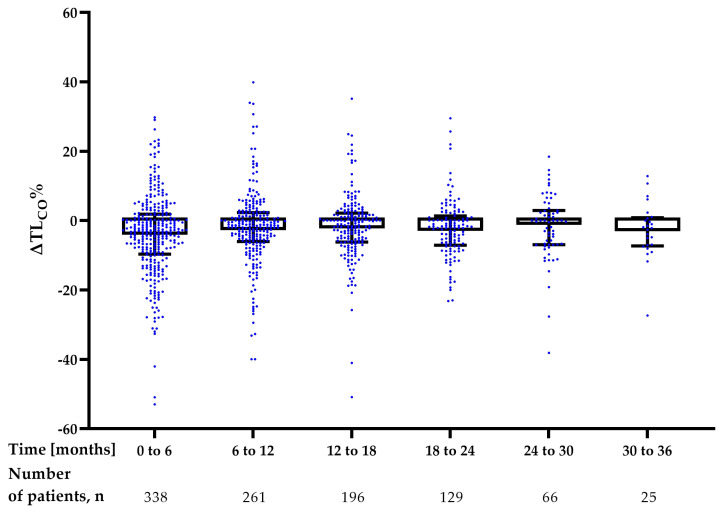
Longitudinal interval changes in TL_CO_% of predicted (ΔTL_CO_%) during nintedanib treatment. Data are presented as median (IQR) values. **Abbreviation:** TL_CO_—transfer factor of the lung for carbon monoxide.

The longitudinal analysis of changes in FVC% of predicted (ΔFVC%) and TL_CO_% of predicted (ΔTL_CO_%) in 6-month intervals is shown in Figure 5 and Figure 6, and Table 4. No significant difference was noted in the rate of decline of FVC% and TL_CO_% in the interval analysis during 36 months of nintedanib therapy.

The additional 6-month interval subanalysis of FVC and TL_CO_ data is shown in Table 5.

**Figure 7:**
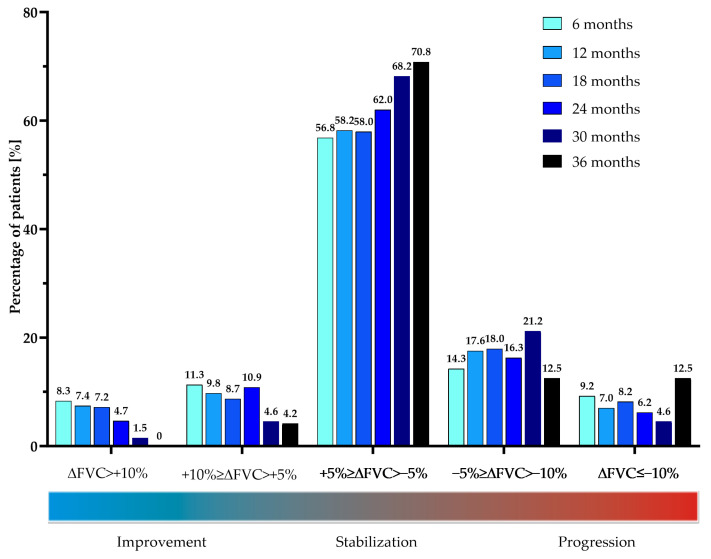
The proportion of patients experiencing significant (ΔFVC > 10%) or marginal (10% ≥ ΔFVC > 5%) improvement, stabilization (+5% ≥ ΔFVC > −5%), and marginal (−5% ≥ ΔFVC > −10%) or significant (ΔFVC ≤−10%) decline based on the rate of changes in FVC% of predicted (ΔFVC%) in consecutive 6-month intervals during nintedanib treatment. **Abbreviation:** FVC—forced vital capacity.

**Figure 8:**
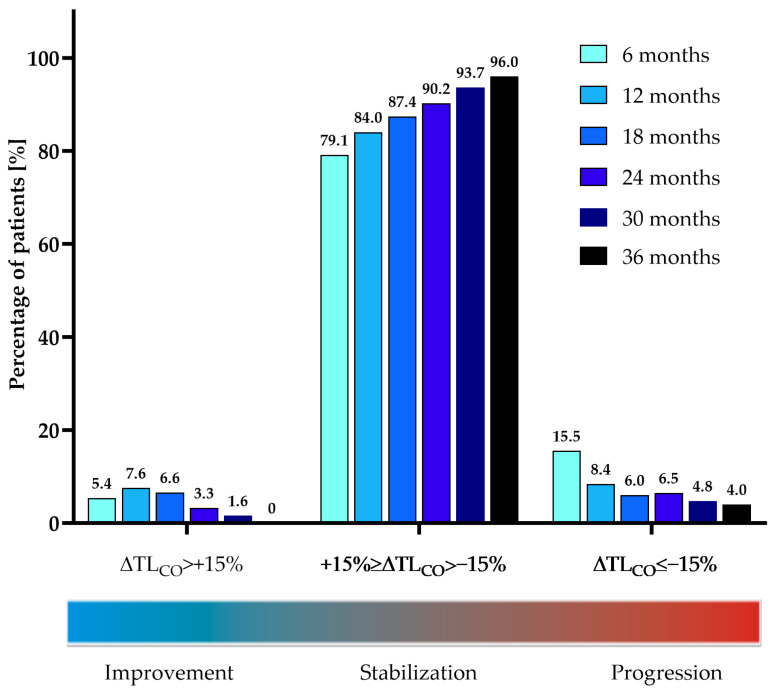
The proportion of patients experiencing significant (ΔTL_CO_ > 15%) improvement, stabilization (+15% ≥ ΔTL_CO_ > −15%), and significant (ΔTL_CO_ ≤ −15%) decline based on the rate of changes in TL_CO_% of predicted (ΔTL_CO_%) in consecutive 6-month intervals during nintedanib treatment. **Abbreviation:** TL_CO_—transfer factor of the lung for carbon monoxide.

Longitudinal changes in FVC% and TL_CO_% over study follow-up varied among the individual patients in our study. Change in FVC% in the majority of patients (range of 56.8–70.8% depending on the interval analyzed) was stable, and only in the minority of them (range 4.2–11.3% and 0–8.3%) showed marginal or significant improvement, respectively. A marginal or significant decline was also observed only in the minority of patients (range 12.5–21.2% and 4.6–12.5%, respectively). In terms of change in TL_CO_% values, longitudinal evaluation confirmed stabilization in the majority of patients (range 85–90.8% depending on the interval analyzed) and a significant improvement or significant decline only in the minority of subjects (range 1.6–5.7% and 4–15.5%, respectively). The graphical presentation of the interval subanalysis of FVC% and TL_CO_% data is shown in Figure 7 and Figure 8.

## 4. Discussion

PolExNIB is the first retrospective and observational study evaluating the long-term safety and efficacy of nintedanib in Polish patients with IPF and is one of the largest multicenter real-world setting studies reporting nationwide experiences of nintedanib in IPF published to date [11,12,13,14,15,16,17,18,19,20,21,22,23,24,25]. The main findings of the present study confirm the long-term acceptable safety and tolerability profiles of nintedanib and suggest that nintedanib therapy is associated with disease stabilization and a low discontinuation rate over up to 3 years of follow-up. The PolExNIB study is a valuable source of evidence on nintedanib treatment in IPF collected from a large representative real-world clinical practice population of patients and a complementary set of data to the recently published work on the Polish experience with pirfenidone therapy in patients with IPF [26]. In addition, our findings are in concordance with the results of previous RCTs and smaller RWD studies on nintedanib in IPF.

Nintedanib is an oral tyrosine kinase inhibitor with antifibrotic and antiangiogenic activity. The mechanism of action of nintedanib in IPF is associated with the inhibition of profibrotic mediators including platelet-derived growth factor (PDGF) and fibroblast growth factor (FGF), as well as a vascular endothelial growth factor (VEGF), which as a result reduces fibroblast activity [31]. The pivotal RCTs including phase 2 (TOMORROW) and replicate phase 3 studies (INPULSIS-1 and INPULSIS-2) confirmed the safety and efficacy of nintedanib and led to drug registration in IPF [6,32]. To date, nintedanib and pirfenidone are the only two drugs currently conditionally recommended for the treatment of IPF by the international clinical practice guidelines [7].

The inclusion criteria for nintedanib therapy in Poland are similar to those applied in the registration studies for nintedanib, namely the INPULSIS trials [6]. RWD studies, like PolExNIB, provide important insight into the value of treatments in clinical practice and supplement data collected in RCTs, and may help answer questions that may not be addressed otherwise [33].

The baseline demographic characteristics of the patients reported in the PolExNIB study, including age, sex, and smoking history, as well as baseline clinical characteristics including lung physiologic parameters (FVC, TLco) were, in general, similar to those reported in the INPULSIS studies [6]. However, the PolExNIB cohort reflected a broader IPF patient population more typical of everyday clinical practice than the INPULSIS cohort limited by the strict inclusion and exclusion criteria. Our cohort of patients presented many comorbidities with cardiovascular system comorbidities being the most frequent, including hypertension (n = 313, 62%), coronary artery disease (n = 128, 25%), and atrial fibrillation (n = 32, 6%). About 13% of participants had a previous history of myocardial infarction (n = 66) and heart failure (n = 65), and almost 7% (n = 34) were receiving oral anticoagulants and another 2% were taking acetylsalicylic acid with oral anticoagulants (n = 9). It is of note that the INPULSIS studies specifically excluded patients with a history of myocardial infarction within 6 months or unstable angina within 1 month before study entry. Moreover, patients at known risk of bleeding including those on full-dose anticoagulation or high-dose anti-platelet therapy were not enrolled in the INPULSIS trials as, based on the mechanism of inhibition of the VEGF receptor, nintedanib may increase the risk of bleeding [34]. An additional comparison between the PolExNIB and INPULSIS cohorts concerning the burden of comorbidities is impossible since comorbidities data were not published in the INPULSIS trials’ final report [6].

The PolExNIB study provided data for up to 3 years of nintedanib treatment, with a median time of 15 months of observation, which is longer than the duration of the INPULSIS trials and several smaller RWD studies on nintedanib in IPF published to date [6,11,12,13,15,16,17,18,19,22,25]. Our data show that nintedanib treatment was safe and associated with acceptable tolerance. As expected based on the results of the previous studies of nintedanib in IPF, the most frequent ADRs were gastrointestinal with diarrhea as the most common event (n = 227, 45%). No new safety signals were identified over the long-term observation. Interestingly, diarrhea was noted less frequently than in the INPULSIS trials (45% vs. 62%). A similar frequency of diarrhea was noted in two other RWD studies, one from Greece (45%) and one from the UK (49.7%) [12,21]. However, the frequency of diarrhea in other real-world clinical setting studies on nintedanib in IPF varies considerably between 32% and 78.9% [11,12,13,14,15,18,19,20,21,22,23,24,25], and is invariably related to differences in the methodologies for collecting data in retrospective studies. Diarrhea was also a main reason for permanent treatment discontinuations due to ADRs in our study (n = 16, 3.2%) which is in complete agreement with the known tolerance profile of nintedanib [6,14,22,35]. It is of note that in INPULSIS-1 diarrhea was a reason for premature discontinuation in 4.5% of patients and in INPULSIS-2 in 4.3% of patients [6]. Other gastrointestinal ADRs noted in our study included decreased appetite, weight loss, abdominal discomfort, nausea, and hepatic enzyme elevations which is in line with the findings on the safety profile of nintedanib reported previously [6,11,12,13,14,18,19,20,21,22,23,24,25,35].

Bleeding was noted only in 4% of patients (n = 20) in our cohort and all bleeding events were not serious. The most frequent were epistaxis and rectal bleeding. Only two patients in our cohort permanently discontinued nintedanib due to bleeding episodes. A lower incidence of bleeding in the PolExNIB compared to the INPULSIS trials (10.2%) could result from the fact that most bleeding events were not severe enough for a patient to seek medical attention and could not be captured in the retrospective study setting. Nevertheless, it is reassuring that the treatment with nintedanib in patients receiving oral anticoagulants is safe as there was only 1 out of 43 patients on oral anticoagulants in our study in whom a bleeding episode (rectal bleeding) was noted during nintedanib treatment. Therefore, our data support the safety of nintedanib therapy in patients at high risk of bleeding including those receiving oral anticoagulants with or without anti-platelet drug. These findings are in complete agreement with the results of the recent analysis of the large cohort of patients with IPF from the European MultiPartner IPF Registry (EMPIRE) showing that bleeding incidence was low during antifibrotic treatment, and irrespective of anticoagulant or anti-platelet therapy received [36].

The majority of patients (66.7%) from the PolExNIB cohort were treated with the full recommended dose of nintedanib, 150 mg b.i.d., and one-third of patients were treated with a reduced dose of the drug, 100 mg b.i.d., due to different tolerance issues and ADRs. Similar chronic dosing of nintedanib was noted in the recent smaller multicenter real-world study in the Greek cohort of patients with IPF [21]. We have not collected data on the reasons or duration of intermittent dose reductions or drug interruptions; nevertheless, temporal dose reductions or drug interruptions were needed in 20% of patients from our cohort. It is a slightly lower rate than reported in the INPULSIS trials [6] and validates that ADRs related to nintedanib treatment are manageable in everyday clinical practice. Moreover, permanent treatment discontinuations due to ADRs were noted in only 10% of subjects in our cohort and were seen less frequently than in the INPULSIS studies (INPULSIS-1 21% and INPULSIS-2 18.8%) but a similar rate was reported in another recent real-world setting study from Greece (13.1%) [6,21]. However, permanent discontinuation rates due to ADRs noted in other RWD studies are usually slightly higher, e.g., 16.1% in the study from the UK [12] or 26% in the study from the USA [13].

On account of the retrospective design of the PolExNIB study, it has not included any specific survival analysis. However, we collected data on the available causes of death during a study follow-up. The main causes of death included disease progression, next to neoplastic disease, and cardiovascular diseases which are in concordance with the findings of other observational studies [37]. About 15% of deaths during the PolExNIB study follow-up were related to coronavirus disease 2019 (COVID-19). This finding supports the view that patients with interstitial lung disease including IPF appear to be more susceptible to infection with severe acute respiratory syndrome coronavirus 2 (SARS-CoV-2), which causes COVID-19, and are at increased risk of severe disease and death when they do contract the disease [38,39]. Unfortunately, our study did not provide data on the prevalence and severity of SARS-CoV-2 infections among all enrolled patients in our cohort.

Over up to 3 years of the study follow-up period, patients’ PFT data remained largely stable. The median annual FVC decline in our study was −60 mL in the first and second years, and −100 mL over the third year. The noted annual rate of decline in our study over the first 2 years was smaller than the annual decline noted in the pivotal RCTs. In INPULSIS-1, the decline was −114.7 mL per year and, in INPULSIS-2, the decline was −113.6 mL per year in the nintedanib group [6]. Moreover, in the PolExNIB cohort, the median change from baseline in FVC% was −1.67% at month 12, −3.08% at month 24, and −5.5% at month 36 which is far below the threshold of a 10% decline in FVC regarded as a marker of significant disease progression [27]. The same observation was noted for the median change from baseline in TL_CO_% of −4.77% at month 12, −6.78% at month 24, and −13.3% at month 36 which is also lower than the clinically significant deterioration threshold of 15% decline [27]. Our study longitudinal assessment of PFT changes over 3-year follow-up performed in 6-month intervals confirmed that the majority of patients obtained disease stabilization or improvement under nintedanib treatment and only a minority of them declined in terms of PFT results. The above findings are in line with the results of the recent real-world setting study on nintedanib in a Greek cohort of patients with IPF which has also shown stable lung function for up to 3-year follow-up [21].

The decline in the 6MWT distance over the PolExNIB study follow-up was optimistic. We noted a median change of only −10m after the first year, −20 m after 2 years, and −45 m after 3 years from the start of nintedanib treatment. However, the number of patients with available data for longitudinal 6MWT analysis was low in the present study. It should be stressed that the longitudinal assessment of the 6MWT distance has been used to reflect the disease status and progression [40,41,42], and prognosis prediction [43]. Moreover, 6MWT decline has been shown to outweigh other predictors of mortality in patients with IPF [44]. A threshold for minimum clinically important difference value for 6MWT has been suggested as 24–45 m or more [45]. Therefore, our data on 6MWT variability over the study follow-up suggest disease stabilization on nintedanib treatment. Neither the INPULSIS trials publication nor RWD studies of heterogenous IPF populations provided data on the longitudinal 6MWT distance changes during nintedanib therapy.

Our study has several strengths. The crucial strengths of the PolExNIB study are the non-sponsored, multicenter construction and evaluation of data collected from one of the largest real-world cohorts of patients with IPF published to date. The median nintedanib exposure and longitudinal observation were also longer than in pivotal RCTs and many of the smaller RWD studies found in the literature. Lack of strict enrollment criteria typical for RCTs allowed for gathering data more representative of the everyday clinical practice population of patients. The broader patient population and a longer follow-up period may likely record a more complete picture of nintedanib safety and efficacy profiles in IPF compared with the RCT data.

The PolExNIB study is not free from limitations inherent in RWD studies. Several possible biases are introduced due to real-world data documentation that is not as robust as that of RCTs. The difference in clinical practices of the participating centers of this multicenter study could lead to missing data and reporting bias. The study efficacy data could be strengthened by pre- and post-treatment comparison of the PFT results, although this was impossible due to a small number of patients with available pre-treatment PFTs. No complete data are available for causes of death due to loss of follow-up for several patients. Moreover, patients discontinuing treatment were excluded from longitudinal study follow-up; therefore, some of our data is incomplete and/or biased concerning patients who took part in regular follow-up visits and who survived for longer periods. The retrospective character of the study did not allow for assessing the possible influence of comorbidities and concomitant medications on the natural history of IPF and nintedanib therapy outcomes. Despite the above limitations, our real-world findings extend the current knowledge on the long-term safety and efficacy profiles of nintedanib in IPF and supplement data obtained from RCTs.

## 5. Conclusions

To sum up, the PolExNIB study provides the first real-world setting data on the safety and efficacy of nintedanib in a large cohort of Polish patients with IPF. Our findings confirm the long-term acceptable safety and tolerability profiles of nintedanib and suggest that nintedanib therapy is associated with disease stabilization and a low discontinuation rate in everyday clinical practice over up to 3 years of follow-up. These data are reassuring for patients and their physicians using nintedanib to slow disease progression and improve patient outcomes. Moreover, the PolExNIB data are aligned with the findings of RCTs and other smaller RWD studies on nintedanib in IPF and support their conclusions.

## Figures and Tables

**Table 1:** Characteristics of study participants.

Number of Patients, n (%)	501 (100)
City of Bydgoszcz, n (%)	26 (5.2)
City of Cracow (2 centers), n (%)	57 (11.4)
City of Gdansk, n (%)	36 (7.2)
City of Katowice, n (%)	13 (2.6)
City of Lodz (2 centers), n (%)	55 (11)
City of Lublin, n (%)	22 (4.3)
City of Olsztyn, n (%)	23 (4.6)
City of Poznan (2 centers), n (%)	61 (12.2)
City of Szczecin, n (%)	25 (5)
City of Warsaw (2 centers), n (%)	114 (22.7)
City of Zabrze, n (%)	49 (9.8)
City of Zielona Gora, n (%)	20 (4)
Sex, male/female, n (%)	349 (69.66)/152 (30.34)
Age (years), median (IQR)	70.9 (65–75.7)
**Smoking history**	
Never smokers, n (%)	134 (26.7)
Former smokers, n (%)	326 (65.1)
Active smokers, n (%)	41 (8.1)
Pack-years, median (IQR)	28 (20–40)
**Comorbidities**	
Hypertension, n (%)	313 (62.5)
Hyperlipidemia, n (%)	186 (37.1)
Coronary artery disease, n (%)	128 (25.5)
History of myocardial infarction, n (%)	65 (13)
Atrial fibrillation, n (%)	32 (6.4)
Heart failure, n (%)	66 (13.2)
Pulmonary hypertension, n (%)	60 (12)
Gastroesophageal reflux disease, n (%)	134 (26.7)
Diabetes, n (%)	134 (26.7)
Emphysema, n (%)	106 (21.2)
Depression, n (%)	52 (10.4)
Obstructive sleep apnea, n (%)	32 (6.4)
Benign prostate hypertrophy, n (%)	96 (19.2)
Neoplastic disease history, n (%)	55 (11)
Osteoarthritis, n (%)	149 (29.8)
Hypothyroidism, n (%)	46 (9.2)
**Concomitant medications**	
Antihypertensive drugs, n (%)	330 (65.9)
Acetylsalicylic acid monotherapy, n (%)	160 (31.9)
Anticoagulants, n (%)	34 (6.8)
Acetylsalicylic acid + anticoagulant therapy, n (%)	9 (1.8)
Proton-pump inhibitors, n (%)	163 (32.5)
Statins, n (%)	205 (40.9)
Antidiabetic medications, n (%)	116 (23.1)
**HRCT pattern**	
Radiologic UIP pattern, n (%)	419 (83.6)
Radiologic probable UIP pattern (%)	68 (13.6)
Radiologic inconsistent for UIP pattern, n (%)	12 (2.4)
**Lung biopsy procedures**	
TBLC, n (%)	18 (3.6)
SLB, n (%)	38 (7.6)
**BAL procedure,** n (%)	102 (20.7)
**Time from first symptoms to diagnosis** (months), median (IQR)	12 (6–28)
**Time from diagnosis to start of nintedanib therapy** (months), median (IQR)	3 (1–11)
**Pulmonary function**	
FVC (l), median (IQR)	2.8 (2.3–3.5)
FVC (% of predicted), median (IQR)	80.2 (68.5–95)
TL_CO_ (mmol/min/kPa), median (IQR)	4.1 (3.2–5.3)
TL_CO_ (% of predicted), median (IQR)	55.9 (42.2–69)
**Blood oxygenation**	
SpO_2_ at rest (%), median (IQR)	94 (91–96)
**6MWT** (n = 288)	
Distance (meters), mean (SD)	436.8 (102.3)
Desaturation, (∆%), median (IQR)	5 (2–8)
**GAP score**, median (IQR)	3 (3–4)
**GAP index**	
Stage I, n (%)	294 (58.9)
Stage II, n (%)	178 (35.7)
Stage III, n (%)	27 (5.4)
**Oxygen therapy**	
Home oxygen therapy, n (%)	69 (13.8)
Portable sources of oxygen, n (%)	26 (5.2)
**IPF treatment in the past before initiation of nintedanib**	
No treatment, n (%)	377 (75.2)
Pirfenidone, n (%)	88 (17.6)
CS, n (%)	41 (8.2)
NAC, n (%)	1 (0.2)
CS + NAC + AZA, n (%)	4 (0.8)
Clinical trial, n (%)	7 (1.4)

**Abbreviations:** UIP—usual interstitial pneumonia, SLB—surgical lung biopsy, TBLC—transbronchial lung cryobiopsy, BAL—bronchoalveolar lavage, FVC—forced vital capacity, IPF—idiopathic pulmonary fibrosis, TL_CO_—transfer factor of the lung for carbon monoxide, SpO_2_—percutaneous oxygen saturation, 6MWT—six-minute walk test, GAP—gender, age, and 2 physiology variables (FVC and TL_CO_), CS—corticosteroids, NAC—N–acetylcysteine, AZA—azathioprine.

**Table 2:** Tolerability and adverse drug reactions (ADRs) of nintedanib treatment.

Full dose treatment 150 mg b.i.d., n (%)	334 (66.7)
Reduced dose treatment 100 mg b.i.d., n (%)	167 (33.3)
Intermittent drug interruption and/or dose reduction, n (%)	101 (20.2)
ADRs	
Diarrhea, n (%)	227 (45.3)
Nausea, n (%)	104 (20.8)
Vomiting, n (%)	41 (8.2)
Abdominal discomfort, n (%)	148 (29.5)
Decreased appetite, n (%)	150 (29.9)
Weight, n (%)	161 (32.1)
Fatigue, n (%)	96 (19.2)
Increased levels of AST/ALT, n (%)	77 (15.4)
Hepatotoxicity AST/ALT > 3 ULN, n (%)	31 (6.2)
Bleeding, n (%)	20 (4)

**Abbreviations:** ADRs—adverse drug reactions, b.i.d.—twice a day, AST—aspartate aminotransferase, ALT—alanine aminotransferase, ULN—upper limit of normal.

**Table 3:** Treatment persistence.

Nintedanib exposure, (months), median (IQR)	15 (7–25.5)
Reasons for treatment discontinuation	
ADRs, n (%)	51 (10.2)
Disease progression, n (%)	23 (4.6)
Death, n (%)	58 (11.6)
Patient’s decision, n (%)	33 (6.6)
Lung transplantation, n (%)	2 (0.4)
Neoplastic disease, n (%)	11 (2.2)
Other, n (%)	25 (5)

**Abbreviations:** ADRs—adverse drug reactions.

**Table 4:** Longitudinal interval changes in FVC% of predicted (∆FVC%) and TL_CO_% of predicted (∆TL_CO_%) during nintedanib treatment. Data are presented as median (IQR) values.

	0–6 Months	6–12 Months	12–18 Months	18–24 Months	24–30 Months	30–36 Months
∆FVC%	−0.96 (−4.94–3.51)	−0.8 (−4.9–2.84)	−0.7 (−5.24–2.89)	−0.9 (−4.8–2.99)	−2.05 (−5.37–1.68)	−1.22 (−5.86–2.86)
∆TL_CO_%	−3.18 (−9.69–1.32)	−1.8 (−6.02–2.29)	−1.35 (−6.2–2.17)	−2.04 (−7.09–1.37)	−0.32 (−6.91–2.9)	−2.18 (−7.28–0.84)
		***p* _6–12 vs. 0–6_**	***p* _12–18 vs. 0–6_**	***p* _18–24 vs. 0–6_**	***p* _24–30 vs. 0–6_**	***p* _30–36 vs. 0–6_**
∆FVC%		0.33	0.18	0.25	0.07	0.7
∆TL_CO_%		0.24	0.18	0.83	0.11	0.89

**Table 5:** Classification of patients in relation to longitudinal interval change in FVC% of predicted (ΔFVC%) and TL_CO%_ of predicted (ΔTL_CO_%) over study follow up.

	0–6 Months	6–12 Months	12–18 Months	18–24 Months	24–30 Months	30–36 Months
∆FVC%						
Significant improvement (∆FVC > 10%), n (%)	28 (8.3)	19 (7.4)	14 (7.2)	6 (4.6)	1 (1.5)	0 (0)
Marginal improvement (10% ≥ ∆FVC > 5%), n (%)	38 (11.3)	25 (9.8)	17 (8.7)	14 (10.8)	3 (4.5)	1 (4.2)
Stabilization (+5% ≥ ∆FVC > −5%), n (%)	191 (56.8)	149 (58.2)	113 (58.0)	80 (62.0)	45 (68.2)	17 (70.8)
Marginal decline (−5% ≥ ∆FVC > −10%), n (%)	48 (14.2)	45 (17.6)	35 (17.9)	21 (16.3)	14 (21.2)	3 (12.5)
Significant decline (∆FVC ≤ −10%), n (%)	31 (9.2)	18 (7.0)	16 (8.2)	8 (6.2)	3 (4.5)	3 (12.5)
n	336	256	195	129	66	24
∆TL_CO_%						
Significant improvement (∆TL_CO_ > 15%), n (%)	17 (5.4)	18 (7.6)	12 (6.6)	4 (3.2)	1 (1.6)	0 (0)
Stabilization (+15% ≥ ∆TL_CO_ > −15%), n (%)	250 (79.1)	200 (84)	160 (87.4)	111 (90.2)	59 (93.6)	24 (96)
Significant decline (∆TL_CO_ ≤ −15%), n (%)	49 (15.5)	20 (8.4)	11 (6.0)	8 (6.5)	3 (4.8)	1 (4)
n	316	238	183	123	63	25

**Abbreviations:** FVC—forced vital capacity; TL_CO_—transfer factor of the lung for carbon monoxide.

## Data Availability

The datasets analyzed during the current study are available from the corresponding author upon reasonable request.
